# Invertebrate Models of Kallmann Syndrome: Molecular Pathogenesis and New Disease Genes

**DOI:** 10.2174/138920213804999174

**Published:** 2013-03

**Authors:** Elia Di Schiavi, Davide Andrenacci

**Affiliations:** Institute of Genetics and Biophysics, Consiglio Nazionale delle Ricerche (CNR), Naples, Italy

**Keywords:** CeKal-1, DmKal-1, Morphogenesis, Axon branching, Animal models, Extracellular matrix.

## Abstract

Kallmann Syndrome is a heritable disorder characterized by congenital anosmia, hypogonadotropic hypogonadism and, less frequently, by other symptoms. The X-linked form of this syndrome is caused by mutations affecting the *KAL1* gene that codes for the extracellular protein anosmin-1. Investigation of *KAL1* function in mice has been hampered by the fact that the murine ortholog has not been identified. Thus studies performed in other animal models have contributed significantly to an understanding of the function of *KAL1*. In this review, the main results obtained using the two invertebrate models, the nematode worm *Caenorhabditis elegans* and the fruit fly *Drosophila melanogaster*, are illustrated and the contribution provided by them to the elucidation of the molecular pathogenesis of Kallmann Syndrome is discussed in detail. Structure-function dissection studies performed in these two animal models have shown how the different domains of anosmin-1 carry out specific functions, also suggesting a novel intramolecular regulation mechanism among the different domains of the protein. The model that emerges is one in which anosmin-1 plays different roles in different tissues, interacting with different components of the extracellular matrix. We also describe how the genetic approach in *C. elegans* has allowed the discovery of the genes involved in *KAL1*-heparan sulfate proteoglycans interactions and the identification of *HS6ST1 *as a new disease gene.

## INTRODUCTION

### Kallmann Syndrome

 Kallmann Syndrome (KS) is a congenital disorder in which anosmia or severe hyposmia (deficient sense of smell) is combined with hypogonadotropic hypogonadism (HH) and, less frequently, with synkinesia (mirror movements), mental retardation, cryptorchidism, cleft lip and/or palate, unilateral renal agenesis and others symptoms [1-5]. KS is a genetically heterogeneous disease with the majority of the patients occurring as sporadic cases. In the rare familial forms, three different modes of inheritance are known: X chromosome linked (Xp22.31), autosomal dominant (8p 11.23-p11.22), and autosomal recessive [6-8]. 

 Post mortem anatomical analysis of a human foetus [9], studies in chicken embryos [10, 48] and more recently the use of *in vivo* neuroimaging techniques in humans [12, 13] have helped define the embryonic developmental defects that underlie KS main symptoms. Anosmia is the result of an impaired migration and targeting of olfactory sensory axons to the olfactory bulbs [14]. HH is in turn a consequence of the failure of gonadotropin-releasing hormone (GnRH) neurons to migrate into the brain and reach the hypothalamus, with the resulting loss in the production and release of the pituitary hormones FSH and LH, which are necessary for gonadal maturation [9, 15, 16]. 

 The gene responsible for the X-linked form of KS was the first to be identified and was named *KAL1 *[17, 18]. This gene encodes anosmin-1, a secreted protein whose structure consists of a signal peptide at the N-terminus followed by a cysteine-rich (CR) region, a whey acidic protein (WAP) domain, four fibronectin type III (FnIII) repeats and a histidine-rich (HR) region at the C-terminus Fig. (**1**). The WAP domain shows similarities to small proteins with serine protease inhibitor activity [19, 20], while the FnIII repeats show high similarity to motifs present in cell-adhesion molecules (CAMs) such as N-CAM, TAG-1 and L1 [21, 22] and probably mediate cell to cell or cell to the extracellular matrix adhesion*. KAL1* orthologs were identified in several vertebrates, and in invertebrates like *Caenorhabditis elegans* and *Drosophila melanogaster*, but not in mouse [23-25]. The domain organization of anosmin-1 orthologs is peculiar and appears highly conserved in the different species Fig. (**1**), indicating that the protein function is also conserved. The main differences among these proteins regard the number of FnIII repeats, which can vary from two to four, the absence of a HR region in invertebrates and the presence at the C-terminus of the *C. elegans* protein of a glycosyl-phosphatidyl-inositol anchoring site (GPI), which probably mediates the binding of the protein to the cell surface.

* KAL1* is responsible for less than 10% of all KS cases [26]; however, for more than 10 years it remained the only identified gene involved in this syndrome. Since 2003 at least six additional genes have been identified [27]. They are the *fibroblast growth factor receptor type 1* (*FGFR1)* [28, 29], the *fibroblast growth factor 8 (FGF8)* [30], *prokineticin 2 *and its receptor (*PROK2* and *PROKR2)* [31], *chromodomain helicase DNA binding protein-7* (*CHD7*) [32] and *heparan sulfate 6-O-sulfotransferase 1* (*HS6ST1*) [33]. Mutations in *FGFR1* cause autosomal dominant forms of KS [28]. Heterozygous and homozygous mutations in the *FGF8* gene, which encodes a ligand for FGFR1 [34], are responsible of GnRH neuron deficiency with variable olfactory phenotypes in KS patients [30]. *PROK2* and *PROKR2* are involved in digenic form of KS, with patients carrying missense mutations both in *PROKR2 *and *KAL1 *[31] or in *PROK2* and *PROKR2 *[35]. Mutations in *CHD7*, the gene responsible for CHARGE syndrome, were found in a heterozygous condition in a minority of KS patients and it has been suggested that KS may be a mild allelic variant of CHARGE syndrome [32, 36]. The last gene associated with KS to be identified is *HS6ST1*, which encodes a heparan sulphate (HS) modification enzyme [33]. *HS6ST1* mutations were found in KS patients in combination with mutations affecting the *FGFR1* gene [33].

 To date in only about 30% of KS cases a causative gene has been identified. This indicates that large part of KS patients present mutations in unknown genes. In addition, all the studies suggest KS as a disease with a complex and heterogeneous genetics, comprising cases with oligogenic mode of inheritance [37, 38].

### Anosmin-1 Function and the Molecular Pathogenesis of Kallmann Syndrome

 The models of molecular pathogenesis of KS have quickly changed over the past ten years. Summarizing the work on humans and vertebrate models it appears that KS is due to alterations of developmental processes occuring during embryogenesis. The most significant defects are in the growth of olfactory axons and the migrations of GnRH-secreting neurons. However also the sporadic symptoms (e.g. renal agenesis, cleft plate, mirror movements) observed in KS patients could be interpreted as alterations of morphogenesis and development of other tissues/districts.

 The first models of molecular pathogenesis of KS were formulated after the identification of *KAL1*, by Lutz and coworkers [11]. At the molecular level two possible roles for anosmin-1 were proposed: 1) anosmin-1 could be a structural protein required for the development and maintenance of the bulb architecture; 2) it could function as an adhesion molecule mediating the interaction between the olfactory axons and the mitral cell dendrites. 

 Soussi-Yanicostas and coworkers [39, 40] demonstrated that anosmin-1 is an adhesive protein that acts as a substrate for neurite outgrowth for different types of neurons and that its adhesive properties depend on the presence in the matrix of HS and chondroitin sulfate glycosaminoglycans. The presence of a WAP domain has suggested, however, a possible role in the inhibition of serine proteases activity. Indeed a direct molecular interaction has been found between anosmin-1 and the urokinase-type plasminogen activator (uPA), leading to propose a model in which anosmin-1 could modulate uPA serine protease activity [41].

 We now know that most of the genes so far implicated in KS code for extracellular proteins. These proteins play functions in the extracellular environment, either as ligands and receptors for signaling or as matrix components acting as substrates for adhesion, growth and migration.

## KAL1 FUNCTION IN INVERTEBRATE MODELS

 Research on KS is now focused on the identification of additional genes responsible for KS and on understanding the role of the identified genes in the developmental processes disrupted in KS patients. A significant contribution to understand the role played by anosmin-1 *in vivo* and to identify new disease genes came from studies in *C. elegans* and *Drosophila*. The absence of a mouse homolog together with the great advantages offered by invertebrate animal models led different laboratories to focus on: 1) the analysis of the function played* in vivo* by anosmin-1 homologs in these models, 2) the structure-function relationship of the protein and 3) the identification of genetic interactors of anosmin-1.

### Invertebrate *Kal-1* Genes are Involved in Morphogenetic Processes and Axonal Outgrowth

 The* kal-1* gene in *C. elegans* encodes a protein (CeKAL-1) of 700 amino acids, composed of a signal peptide, a cysteine-rich domain, a WAP-type protease inhibitor domain, three FNIII repeats and a predicted GPI-anchoring site Fig. (**1**). The protein is 30% identical and 50% similar to human anosmin-1 [23]. The expression of the gene in the worm is detectable in a subset of neuroblasts during embryogenesis [23, 24, 42]. Post-embryonically, three groups of neurons express *kal-1 *Fig. (**2a**): one group is located in the anterior ganglia, a second group at mid body and a third group in the tail region. A deletion mutant was generated by chemical mutagenesis and this null mutant is at the moment the only animal model with a complete loss of function of the *kal-1* gene and therefore an unique tool to investigate its function *in vivo*. Loss of function animals are apparently normal. Using high-resolution microscopy and a variety of GFP markers that help visualize neurons and epithelial cells it was possible to reveal a variety of subtle but specific defects, including: embryonic development retardation and ventral closure defects, alterations in the morphogenesis of the adult male tail, and neurite outgrowth defects [23, 42, 43]. Similar phenotypes were also induced by overexpression experiments (see below) [24]. Using these models it was found that *kal-1* expression in neurons influences morphogenesis of nearby epidermal cells by regulating their adhesion and migration, while it controls neuron development in a cell-autonomous fashion, modulating neurite outgrowth and branching [23, 24]. Notably the male tail defects of *kal-1 *mutant animals were successfully rescued by expressing the human *KAL1* cDNA under the *C. elegans* *kal-1* promoter [23]. These results indicate that conservation of the anosmin-1 protein between humans and nematodes is not limited to structure, but is also functional and gives further support to the use of *C. elegans* as an animal model to study KS *in vivo*.

 In *Drosophila*, the *kal-1* gene encodes a protein (DmKal-1) shorter than vertebrate and *C. elegans* orthologs, with only two FnIII repeats Fig. (**1**). DmKal-1 is composed of 525 amino acid and shares 23% identity and 36% similarity with the human protein [25]. DmKal-1 expressed in cultured cells is localized on the cellular surface and shows a weak affinity to the cell membrane [44]. The presence of only two FnIII repeat is possibly the cause of the reduced adhesiveness of the protein. *kal-1* shows a complex expression pattern during the second half of embryogenesis and its transcripts are detectable in different epithelial cells involved in the morphogenetic processes of germ band retraction, dorsal closure and head involution as well as in cells associated with different sensory organs including the antenno-maxillary organ, which carries out olfactory and gustatory functions in larvae [25]. Overexpression experiments performed in embryos and in the wing disc indicate that DmKal-1 may be involved in the morphological processes that lead to the formation of the larval mouth and of the adult wing [44].

 The data on nematodes and fruit flies indicate that invertebrate anosmins are involved in axon outgrowth and in epithelial morphogenesis in analogy with what is observed in humans and other vertebrates.

### Structure-Function Analysis of Invertebrate Anosmin-1 Proteins

 Structure-function dissection analysis was used in *C. elegans* and *Drosophila* to better understand the role of the different domains of anosmin-1.

 Overexpression of CeKAL-1 in the AIY interneurons produced highly penetrant and specific AIY axon branching defects Fig. (**2b**) [24]. This extra-branching phenotype was dosage-dependent and was not observed by expressing randomly chosen proteins containing WAP or FnIII domains. A C-terminal deletion of CeKAL-1, eliminating part of the first and all the subsequent three FnIII motifs Fig. (**3a**, ∆C), did not cause any extra-branching when expressed in the same head neurons. A similar loss of activity was observed after expression of CeKAL-1 carrying a single substitution in the first FnIII motif corresponding to the N267K mutation found in human patients Fig. (**3a**, S241K). These results suggest a fundamental role of the FnIII domains in extra-branches formation. On the other hand, the elimination of two disulfide bonds in the WAP domain Fig. (**3a**, mWAP) did not abolish the ability to cause branching alterations but qualitatively changed the phenotypes with a more extensive arborization of the branches. This suggests a possible instructive role of the WAP domain in axon branching. Pan-neuronal expression of CeKAL-1 (not restricted to single head neurons) induced a highly penetrant axon fascicle-misrouting defect in head sensory neurons. The ability to induce this phenotype was maintained by the S241K mutant but was partially lost by the mWAP mutant protein Fig. (**3a**). This was the first experimental evidence of anosmin-1 as a multifunctional protein, with WAP and FnIII domains exerting distinct roles in different processes. However, these roles are not completely separable as the elimination of the FnIII motifs completely abolishes the ability of the protein to induce the axon branching defect, demonstrating that the WAP domain needs the presence of the other domains to carry out its function. 

 A functional dissection was also performed on the *Drosophila* DmKal-1 protein, by analyzing the phenotypes induced by the overexpression of different mutant proteins in the larval cephalopharyngeal skeleton and in adult wing formation [44]. As reported above, overexpression of the wild type DmKal-1 protein induced morphological alterations in the larval head skeleton and in adult wings. Two deletions, either eliminating the two FnIII motifs Fig. (**3b**, W200-STOP) or only the C-terminal region downstream of the second FnIII motifs Fig. (**3b**, H384-STOP), completely abolished the activity of the protein. These experiments demonstrated that not only the FnIII domains, but also the C-terminal region, containing two consensus sequences for HS binding sites, are essential for the function of the protein. The introduction in the DmKal-1 protein of a mutation analogous to the N267K single substitution found in human patients Fig. (**3b**, N236K) caused, in the larval head skeleton and in adult wings, the same phenotype induced by the wild type protein. However, the N236K mutation also induced strong alterations in the head involution morphogenetic process. This result suggests that N236K does not impair the function of the protein but induces a gain of function effect on a different process. Although a change in the overall adhesive properties of the N236K mutant compared to the wild type protein could not be demonstrated, it is possible that this substitution has produced a change in the affinity towards one or more specific interacting substrates. Interestingly, the same mutated protein did not induce similar gain of function effects in the wing, highlighting the importance of the context in which the protein works.

 A double point mutation in the consensus sequence of a non-conserved HS binding site in the first FnIII domain Fig. (**3b**, R159T and R161L) only induces little changes in the protein activity. Proteins carrying point mutations in the CR region Fig. (**3b**, C85S) or in the WAP domain Fig. (**3b**, C127/128S) were still able to induce the same phenotypes as the wild type in the larval head skeleton and in adult wings. When, however, the C127/128S double substitution in the WAP domain was introduced together with the N236K substitution in the same protein, the gain of function effect produced by the mutation in the FnIII motif was suppressed. Thus, as in *C. elegans*, the WAP domain of DmKal-1 seems to be dispensable for some of the functions played by the protein, but its role appears essential for others. These results also suggest that the WAP domain may have a regulatory function on the fibronectin domains and on the adhesive properties of the molecule. This is in agreement with experiments performed in cell culture showing that a mutation in one of the cysteines of the WAP domain interferes with the function of the first FnIII motif [41]. This phenomenon has been explained hypothesizing that structural alterations of the WAP domain might influence the distribution of charged residues in the first FnIII domain.

 The experiments described demonstrate the importance of the FnIII domains for anosmin-1 function, suggesting a regulatory role of the WAP domain and of the CR region on the adhesive property of the FnIII domains. Finally, these experiments suggest that anosmin-1 may act differently in different tissues, probably because of the diverse contributions of the domains in each cellular context.

### *Kal-1* Genetic and Physical Interactors

 In a genetic screening performed in *C. elegans*, several mutations were isolated that selectively suppressed the axon branching phenotype of the AIY neuron induced by CeKAL-1 overexpression [24]. One of these mutations was mapped to the heparan-6-O-sulfotransferase gene (*hst-6*), which encodes an enzyme that catalyzes the transfer of a sulfate moiety to the glucosamine residue in position 6. A second mutation was found in the HS glucuronyl C5-epimerase (*hse-5*), another HS chain modifier enzyme that epimerizes glucuronic acid units to the isomeric iduronic acid [45]. A third mutation was found in the PAPS (3’-phospho-adenosine-5’-phosphosulfate) transporter gene (*pst-1*), a specific carrier for the transport into the Golgi of PAPS, the universal sulfate donor for all sulfotransferases enzymatic reactions [46]. All these genes encode proteins involved in the modification of HS. Mutations in *hse-5 *but not in* hst-6 *suppressed the epidermal defects caused by pan-neuronal expression of *kal-1* [24]. Moreover, genetic interactions using the *kal-1* null mutant were found with C5-epimerized and 6-O-sulfated HS chains attached to proteoglycans during neuroblasts migration [42]. A direct molecular association of CeKAL-1 with syndecan/SDN-1 and with glypican/GPN-1, mediated by the HS chain, was also demonstrated to be necessary to promote cell migration [42]. In summary it appears that the different phenotypes observed in *C. elegans* mutants are dependent on specific combinations of HS modifications, highlighting again the importance of the extracellular context for the function of anosmin-1 [47]. The study on the HS modifying enzymes in *C. elegans* has also opened the way to the discovery that the human ortholog of *hst-6* (*HS6ST1*) is involved in normosmic HH (nHH) and in KS [33].

 After the discovery that *FGFR1* is responsible for an autosomal dominant form of KS, it was hypothesized that anosmin-1 could interact with FGFR to promote the binding of the FGF ligand [28]. Since in *C. elegans* the FGF/FGFR signaling is not involved in ventral neuroblasts migration and no genetic interaction with *kal-1* was found, the involvement of a different unknown pathway to regulate this particular phenotype has been proposed [42]. Nevertheless, a possible interaction between anosmin-1, HS proteoglycans (HSPGs) and the FGF pathway has been investigated in other models. Evidences were found of *KAL1* and *Fgfr1* coexpression in different human tissues [10, 48-50], and *in vitro* analysis demonstrated that anosmin-1 can act as a co-ligand and modulator of the FGFR1/FGF2/HSPGs complex [51, 52]. Hu and coworkers [53] also demonstrated that the domains of anosmin-1 that mediate the interaction with FGFR1 are the first FnIII motif, the CR region and the WAP domains. A weak interaction between anosmin-1 and FGFR2c was also demonstrated, and it required the CR region and the WAP domains but only to have an optimal FGFR binding. These cell culture data confirm that the CR and the WAP domain may have a regulatory role on the function of the FnIII domains, as suggested by the experiments in *C. elegans *and *Drosophila* described above.

*In vivo* confirmation of a functional interaction among anosmin-1/HSPGs/FGF/FGFR was given by genetic studies performed in the *C. elegans* nervous system, looking at a new phenotype [33]. In *C. elegans* only one FGF receptor and one ligand are present, encoded respectively by the *egl-15* and *egl-17* genes. These genes are not involved in CeKAL-1 induced axon branching phenotype of the AIY interneuron. However, a similar branching defect was induced also by the misexpression of CeKAL-1 in the AFD sensory neurons which, differently from AIY, require FGFR/ *egl-15* for their normal development [54]. Also the AFD extra-branching phenotype induced by CeKAL-1 is suppressed by mutations in the HS modifying enzymes [33]. In addition this phenotype was significantly suppressed by loss of FGFR/*egl-15* or of its ligand FGF/*egl-17*. The suppression of the AFD branching phenotype by loss of the ligand was stronger than by that of the receptor but not as complete as that due to the loss of HS. This result is important because it supports the idea that anosmin-1 can interact with other signaling pathways in addition to the FGF/FGFR one. This work offers the first *in vivo* demonstration of the role of anosmin-1 as an important modulator of the interaction among HSPGs/FGF/FGFR and also confirms the existence of other pathways, not involving FGFR, in which anosmin-1 and HSPGs may have a similar modulatory role.

## A NEW MODEL OF MOLECULAR PATHOGENESIS

 It is now clear that KS is a disease mainly involving extracellular interactions. Most of the genes identified so far as involved in KS code for extracellular matrix components or modificators, and for membrane proteins. In the matrix a relatively large number of proteins are present and interact with each other to ensure the correct execution of developmental processes, such as cell and neurite migration. In different districts the combination of proteins present is slightly or significantly different and so are their reciprocal interactions. Although these interactions do not all fall under the ligand/receptor paradigm, the results on anosmin-1 obtained especially with the invertebrate models make it possible to formulate a model for the function of anosmin-1 and of some of the other proteins involved in KS Fig. (**4**). To carry out its function, anosmin-1 must interact with HSPGs through its HS binding sites mainly localized in the FnIII motifs and, in fact, the deletion of the FnIII motifs completely impairs the function of the protein Fig. (**4a**, grey arrow). Sulfation of proteoglycans and glycosaminoglycans influences the interactions of the proteins involved in the developmental processes affected in KS and, accordingly, mutations in the gene encoding the modifying enzymes HS6ST1 were found in nHH and KS patients [33]. This interaction between FnIII motifs and HSPGs is regulated by the CR region and by the WAP domain Fig. (**4a**, black arrow). As it has been shown previously, the disruption of disulfide bonds in the WAP domain may have, however, different effects on the function of the protein depending on the context. The evidence emerged from experiments performed in *C. elegans*, *Drosophila *and in cell culture and is also confirmed by clinical observations: cases of KS patients with mild sexual phenotypes carrying missense mutations that disrupt disulfide bonds in the WAP domain are well documented [55, 56]. The interaction between anosmin-1 and FGFR requires the WAP domain, and probably the CR region [57], which seem to have a regulatory role on the adhesiveness of the FnIII domains present in the same molecule Fig. (**4a**, black arrow). In other extracellular contexts however this regulatory function seems to be dispensable Fig. (**4b**). For example, it is possible that the interaction of anosmin-1-HSPGs with other ligand/receptor complexes may be less dependent on the regulation of the CR region and of the WAP domain Fig. (**4b**). It is interesting to note that the AFD branching phenotype induced by CeKAL-1 was almost completely suppressed by mutations affecting genes involved in HS modification whereas the loss of function of FGF and FGFR only partially inhibited this branching phenotype. This suggests that alterations of AFD axon branching are produced by the simultaneous involvement of at least two different signaling pathways. The interaction of anosmin-1 with other signaling pathways is confirmed by the observation that both the ventral neuroblast migration in which CeKAL-1 is involved [42] and the CeKAL-1 induced AIY axon branching phenotype [24] did not involve the FGF/FGFR pathway. These evidences suggest that anosmin-1 is able to regulate different types of HSPGs/ligand/receptor interactions in different extracellular context.

 Work done in *C. elegans* again suggested which alternative pathway could play a role in KS [42]. *kal-1* null mutation significantly enhanced ventral neuroblasts migration defects and other embryonic phenotypes produced by mutations in the Ephrin, PTP-3 (LAR-related receptor tyrosine phosphatase), Semaphorin and Robo signaling pathways. Since *kal-1* enhances the effect of null mutations in all these genes, it has been suggested that CeKAL-1 may act in parallel with these pathways to carry out its function. Consistently, mice lacking Semaphorins pathway genes showed anatomical defects very similar to those associated to KS [58-60] and two recent papers confirmed *SEMA3a* to be a gene responsible for the disease [61, 62]. Since there are evidences that the biological activity of SEMA3a is enhanced by proteoglycans [63], it is possible that anosmin-1 may directly interact with the *SEMA3a* pathway.

 Another possible involvement of anosmin-1 in a different signal pathway regards the PROK2/PROKR2 pathway. PROK2 have binding affinities for HS-glycosaminoglycans and in addition there is a case of a digenic form of KS with a patient heterozygous for a mutation in *PROKR2* also carrying a missense mutation in *KAL1* [31]. In this case, however, the invertebrate models are not suitable for testing a genetic interaction because orthologs of *PROK2* and *PROKR2* are not present in *C. elegans* and *Drosophila*.

## CONCLUSION

 Kallmann Syndrome is a genetic heterogeneous disorder characterized by two main symptoms, anosmia and hypogonadotropic hypogonadism. Different genes involved in this disease have been identified and their role in the molecular pathogenesis is becoming more clear. In this review we summarized the contribution to the understanding of the molecular pathogenesis of KS provided by the two invertebrate model organisms, *Caenorhabditis elegans* and *Drosophila melanogaster*. 

 We illustrated how structure-function dissection of anosmin-1 in invertebrates has contributed to better understand the function that the different domains play* in vivo*. These experiments determined that anosmin-1 acts differently in different tissues, with the contribution of each domain varying in the various cellular context. The interactions with other components of the extracellular matrix are strictly dependent on HSPGs and on specific combinations of HS modifications, that are different depending on the tissue. In addition, these studies suggest a novel intramolecular regulation mechanism, which has been confirmed by *in vitro* studies. The multidomain function and tissue specificity may be the reason why different sets of phenotypes have been observed in different KS patients [5, 64]. Finally, this complexity may also explains some of the difficulties in finding new disease genes and in strictly correlating genotype and phenotype, even in monozygotic twins [5, 64].

 Genetic screenings carried out in *C. elegans* have been very useful for elucidating, at least partially, the pathways in which anosmin-1 is involved and have contributed to the discovery of a new gene involved in KS [33]. The fact that anosmin-1 has different effects depending on the tissue or cells under analysis suggests that new genetic screenings based on different phenotypes can potentially be useful for identifying new interactors, that can represent new candidate genes for the disorder. Moreover the analysis of *kal-1* expression in *C. elegans* has already been demonstrated to be transcriptionally activated by the homeobox genes *ceh-10/VSX2*, *ttx-3/LHX2* and *lim-4/LHX9 *[66, 67]. Interestingly the mouse homologs of these three genes are all expressed in the olfactory bulb [68, 69] suggesting a conserved regulatory function and strongly candidating these as new potential KS genes.

 The well defined molecular pathways and the availability of powerful genetic techniques in *C. elegans* and *Drosophila* will be very useful to understand the still elusive pathogenesis of KS, in particular in elucidating the complex extracellular interactions that are impaired in KS-affected patients. In addition studies in these models can help to understand the function played *in vivo* by causative genes recently identified in other systems, like *SEMA3a* and to clarify their eventual interactions with established KS genes. Finally, with their simplicity and easy manipulation they can still play a role in identifying new genetic partners, that can represent new candidate genes to be responsible for the remaining 70% of KS cases, whose causative gene is still not known.

## Figures and Tables

**Fig. (1):**
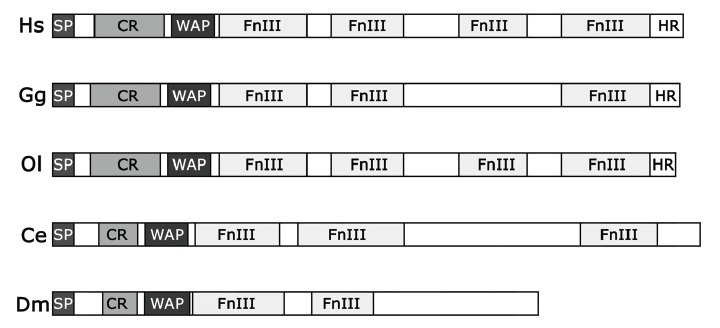
Schematic domain structure of anosmin-1 proteins in *Homo sapiens* (Hs), *Gallus gallus* (Gg), *Oryzias latipes* (Ol), *Caenorhabditis elegans* (Ce) and *Drosophila melanogaster* (Dm). SP, signal peptide; CR, cysteine-rich region; WAP, whey acidic protein domain; FnIII, fibronectin type III repeat; HR, histidine-rich region.

**Fig. (2):**
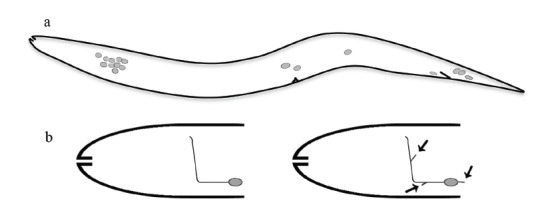
**(a)** Scheme of the cells expressing *kal-1* in *C. elegans*, as identified by a GFP-reporter approach (anterior is to the left, ventral is down). A first group of cells, located in the anterior ganglia (left), is formed by about 15 neurons, including some interneurons and some sensory neurons (e.g. AIY, AIZ, RID, M5, ASI, M5). A second group is located at mid body and is composed of the canal associated neuron (CAN), of the hermaphrodite specific neuron (HSN) and of the posterior ventral mechanosensory neuron PVM. A third group is located in the tail region (right), where three to six neurons express the construct. Among these are the motor neurons PDB and DVB, and the interneurons DVC and PVW. **(b)** Schematic drawing of the head interneuron AIY and its axon in wild type (left) and in *kal-1* overexpressing worms (right), that show extra-branchings (arrows).

**Fig. (3):**
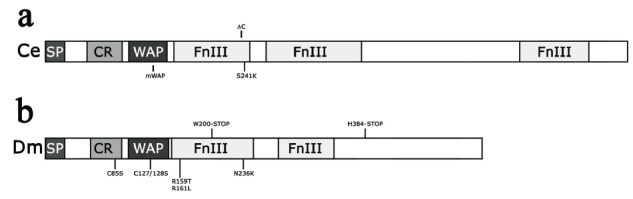
Schematic structure of CeKAL-1 **(a)** and DmKal-1 **(b)** with the respective mutations analyzed.

**Fig. (4):**
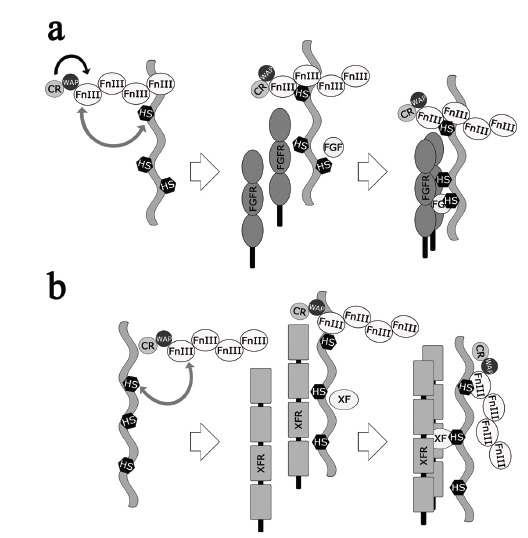
Molecular models of anosmin-1 function. **(a)** From left to right, the WAP domain and the CR region interact with the FnIII repeats (dark arrow) regulating the binding to HS (grey arrow), promoting the interactions with FGFR, FGF, and the formation of the signaling complex. **(b)** From left to right, the binding of the FnIII repeats to HS attached to a different proteoglycan (grey arrow) does not require the WAP domain and the CR region activity. This interaction promotes the formation of a signaling complex with an unknown X-factor **(XF)** and its receptor **(XFR)**. The two models are not alternative but represent the possible functioning of anosmin-1 in different contexts.
